# Two different genotypes of Vibrio parahaemolyticus serotype O10:K4 have been co-circulating in Shanghai since 2020

**DOI:** 10.1099/mgen.0.001648

**Published:** 2026-02-16

**Authors:** Chaonan Wang, Wanju Zhang, Qingneng Lin, Yong Chen, Hui Jiang, Jing Zhou, Bowen Li, Peixuan Li, Yuan Zhuang, Min Chen, Hongyou Chen

**Affiliations:** 1 Microbiology Department, Shanghai Municipal Center for Disease Control and Prevention, Shanghai, 201107, PR China

**Keywords:** genome, genotype, serogroup, serotyping, *Vibrio parahaemolyticus*

## Abstract

In this study, we conducted serotyping, virulence gene detection and whole-genome sequencing on *Vibrio parahaemolyticus* strains collected from the Shanghai area between 2017 and 2021. A total of 369 genomic sequences of different serotypes were obtained, including 155 serotype O10:K4 strains. It was found that the O10:K4 serotype presents two subtypes (O10:K4_*α*_ and O10:K4_*β*_) co-circulated. They were both pandemic strains. Since 2020, the serotype O10:K4 has become the new dominant serotype in the Shanghai area. Among them, the O10:K4_*α*_ subtype first appeared in the Shanghai area in 2019, while the O10:K4_*β*_ subtype first appeared in 2020. However, the O10:K4_*β*_ subtype has a broader range of prevalence than the O10:K4_*α*_ subtype. Upon comparison, it was found that both the O10:K4_*α*_ subtype and the O10:K4_*β*_ subtype carry different unique genetic islands, and the compositions of the O antigen-determining region are also different. The O antigen-determining region has low similarity with the O10 group of *V. parahaemolyticus*, which is closer to the non-epidemic strain O4:K4. The SNP phylogenetic tree analysis revealed that the O10:K4_*α*_ subtype has a close genetic relationship with the O10:KUT serotype strains, while the O10:K4_*β*_ subtype has a closer relationship with the O10:K60 serotype strains, which is consistent with the results of core genome multilocus sequence typing analysis using the public database of *V. parahaemolyticus* genomes. The emergence of these two novel serotypes of *V. parahaemolyticus* is still in constant flux, so enhancing surveillance for *V. parahaemolyticus* is crucial for the prevention and control of foodborne illnesses it may cause.

**Impact Statement**

This study identified the pandemic strain O10:K4, which first emerged in Shanghai, China, in 2019. To further explore the epidemiological and molecular characteristics of the O10:K4 serotype, we analysed epidemiology and genetic diversity of *Vibrio parahaemolyticus* in Shanghai, China, between 2017 and 2021. By whole-genome sequencing combined with serotyping analysis, we identified that the pandemic strain O10:K4 is characterized by its two genetic subtypes, O10:K4_*α*_ and O10:K4_*β*_. Notably, although O10:K4_*β*_ was discovered later in 2020, it rapidly surpassed O10:K4_*α*_ in quantity and achieved a broader epidemic range. Crucially, the two subtypes exhibit unique compositions in the O antigen determinant region, making a departure from previously discovered O10 groups. Our exploration of their origins provided a basis for tracking the evolution of *V. parahaemolyticus*, facilitating more precise molecular epidemiological investigations. These findings underscore the necessity of integrating genomic surveillance into regional public health strategies, which can guide the design of PCR assays targeting antigenic regions, optimize preventive interventions and enhance preparedness for epidemic response. The O10:K4_*α*_ and O10:K4_*β*_ genotypes circulating in Shanghai have distinct evolutionary origins, which may be associated with horizontal gene transfer events or the insertion of exogenous genetic elements. Therefore, it is highly necessary to conduct whole-genome sequencing of *V. parahaemolyticus* and obtain information about its potential origin through genomic sequence analysis. This process is also essential for tracing the source of *V. parahaemolyticus* and acquiring characteristics of its antigenic diversity.

## Data Summary

The authors confirm that all supporting data, code and protocols have been included in the article or provided via supplementary data files. All Illumina and MGI sequencing reads have been submitted to NCBI under the study project PRJNA1213288 (accession numbers listed in Table S1).

## Introduction

*Vibrio parahaemolyticus* is a halophilic Gram-negative bacillus, which is widely found in marine environments and estuaries and is one of the most important pathogenic agents causing acute gastroenteritis in humans [1,2]. Most strains of *V. parahaemolyticus* carry a single haemolysin gene, while a minority carry two virulence genes for haemolysins that are thermostable direct haemolysin (*tdh*) and *tdh*-related haemolysin (*trh*) [3]. The serotypes of *V. parahaemolyticus* are determined by the combination of lipopolysaccharide (LPS) heat-stable somatic antigen (O antigen) and the capsular antigen (K antigen), encompassing a total of 13 different O antigen groups and 71 different K antigen types [4]. The variations in LPS heat-stable somatic and capsular antigens may enhance their resistance to adverse environmental factors and digestive fluids in the human gastrointestinal tract, making them more likely to gain a competitive advantage in the environment and thus accumulate in large quantities in food [5].

*V. parahaemolyticus* was first isolated during a food poisoning incident in Osaka, Japan, in 1950, which was caused by sardines. Subsequently, food poisoning incidents caused by *V. parahaemolyticus* have been reported worldwide [6]. Since 1996, the O3:K6 serotype and its serological variants, such as O4:K68, O1:K25 and O1:KUT, caused a pandemic worldwide, with outbreaks and/or sporadic cases of diarrhoea caused by them reported in numerous countries [7,8]. These isolates are referred to as pandemic strains, all of which carry specific mutation (*toxRS-Mut*) with virulence genes *tdh* but not *trh*. Although the core genome composition of the O3:K6 serotype and its serotype variants is almost identical, there are significant differences in their antigen-encoding gene clusters, which allow for the derivation of a variety of serotype variants [9,11]. At the same time, a series of new gene clusters encoding different heterologous antigens is inserted into the genome of *V. parahaemolyticus*, thereby significantly enhancing its pathogenicity. *V. parahaemolyticus* was isolated and identified from the faeces of patients with foodborne diseases or diarrhoea in Shanghai, China, in 1957. Since the 1990s, *V. parahaemolyticus* has become a major pathogen causing foodborne illnesses and infectious diarrhoea in China [12]. In 2002, the serovar O3:K6 of *V. parahaemolyticus* became the predominant serovar in Zhejiang Province, China, and subsequently, it was confirmed as a pandemic strain clone in 2008 [13]. Between 2007 and 2012, ~56% of clinical isolates from the southern coastal areas of China exhibited pandemic strain characteristics, with the most predominant serovar being O3:K6, followed by O4:K8 and O3:K29 [12]. It’s worth noting that a food poisoning incident caused by the O10:K4 serotype of *V. parahaemolyticus* was first reported in 2020 in the Guangxi Zhuang Autonomous Region of China [14]. After being detected in Huzhou City, Zhejiang Province, in 2020, it became the dominant circulating serotype in 2021, surpassing that of the previously dominant serotype O3:K6 [15]. Subsequently, outbreaks involving the same serotype O10:K4 have also occurred in Beijing, China, and Thailand [16,17].

In this study, we found that the O10:K4 serotype first emerged in the Shanghai area in 2019. We have observed that since 2020, serotype O10:K4 has emerged as a new predominant strain in the Shanghai region, co-circulating as two distinct subtypes: O10:K4_*α*_ and O10:K4_*β*_. Notably, the O10:K4_*β*_ subtype exhibited a broader range of prevalence compared to the O10:K4_*α*_ subtype. Through whole-genome sequencing analysis, we determined that the O10:K4 serotype of *V. parahaemolyticus* prevalent in Shanghai can be divided into two genetic subtypes, and we investigated the possible origins of the two subtypes. Using next-generation sequencing, we obtained a total of 369 genomic sequences of *V. parahaemolyticus*. Our analysis revealed significant differences between the two subtypes in terms of their unique genomic islands (GIs) and the composition of the O antigen determination regions.

Interestingly, we found that the nucleotide sequences of the O antigen determination region in the O10:K4_*β*_ subtype showed higher similarity to those of the non-epidemic strain O4:K4 rather than to its counterpart, O10:K4_*α*_. Both core genome multilocus sequence typing (cgMLST) and SNP analyses indicated that the two O10 subtypes circulating in Shanghai have distinctly evolutionary origins, which may be associated with horizontal gene transfer events and the insertion of exogenous genetic material.

## Methods

### *V. parahaemolyticus* collection and serotyping

Isolates were collected through 16 different districts of Shanghai hospitals’ surveillance from patients with acute diarrhoea or seafood products from municipal markets during 2017–2021 and sent to the Shanghai Municipal Center for Disease Control and Prevention. Chromogenic Vibrio Agar was used to isolate *V. parahaemolyticus* strains, then a single colony was picked out to culture with Tryptic Soy Agar (TSA) containing 3% NaCl. Serotyping of *V. parahaemolyticus* strains was determined by a slide agglutination test for 11 O (LPS) and 65 K (capsule) antisera (Denka Seiken Ltd., Tokyo, Japan) according to the manufacturer’s instructions. One serotype was defined as a unique combination of O and K serogroups.

### Detection of virulence-associated genes

The whole genomic DNAs of the strains were prepared in TE buffer (pH 8.0), bathed at 100 °C for 10 min and centrifuged at 10,000 r.p.m. for 10 minutes. The DNAs were stored at −20 °C until use. PCR [11] was performed for *tdh*, *trh*, *toxRS-Mut*, *toxRS* and *orf8* detection with their primers used in this study listed in Table 1. For *tdh*, *trh* and *orf8*, the PCR mixture was heated at 94 °C for 3 min prior to 30 cycles of PCR amplification in a DNA thermal cycler; one PCR cycle consisted of denaturation at 94 °C for 30 s, primer annealing at 52 °C for 1 min and extension at 72 °C for 1 min; after the last cycle, the PCR mixtures were incubated at 72 °C for 10 min. For *toxRS-Mut* and *toxRS*, the PCR mixture was heated at 94 °C for 3 min, 25 cycles consisted of 94 °C for 30 s, 47 °C for 2 min and 72 °C for 2 min, then the PCR mixtures were incubated at 72 °C for 7 min. The PCR products were electrophoresed in 1.0% agarose gels and stained with ethidium bromide visualized by UV illumination.

**Table 1.** PCR primers used in this study

Primer name	Sequence (5′-3′)
*tdh*_F	GTAAAGGTCTCTGACTTTTGGAC
*tdh*_R	TGGAATAGAACCTTCATCTTCACC
*trh*_F	TTGGCTTCGATATTTTCAGTATCT
*trh*_R	CATAACAAACATATGCCCATTTCC
*toxRS-Mut*_F	TAATGAGGTAGAAACA
*toxRS-Mut*_R	ACGTAACGGGCCTACA
*toxRS*_F	TAATGAGGTAGAAACG
*toxRS*_R	ACGTAACGGGCCTACG
*orf8*_F	GTTCGCATACAGTTGAGG
*orf8*_R	AAGTACAGCAGGAGTGAG

### Whole-genome sequencing

The DNA was extracted from pure culture on TSA containing 3% NaCl with Qiagen QIAamp DNA Mini Kit (QIAGEN Cat. No. 51304, Germany) according to the manufacturer’s instructions, and DNA quality control was performed using agarose gel electrophoresis and the Qubit dsDNA HS Assay Kit. The TE buffer (pH 8.0) supernatant contained strain DNA, which was collected and stored at −20 °C until use. Whole-genome sequencing of strains was performed with libraries constructed using MGIEasy Fast FS Library Prep Set on MGI Tech (Wuhan, China) and those with Illumina DNA Prep kit on Illumina (USA). Ligation Sequencing Kit (SQK-LSK109) and Oxford Nanopore (England) platform were used for supplementation.

### *De novo* genome assembly and genome annotation

The draft genome was assembled *de novo* with SPAdes version 3.15.5 [18], and the contigs of these lengths <1,000 bp and depth <10× were cut off. CheckM version 1.2.2 [19] was used to assess the quality of these assemblies, and only assemblies with completeness over 95% and contamination under 5% were kept. Afterwards, QUAST version 5.2.0 [20] was also used to assess the quality of the assemblies. Prokka version 1.14.6 [21] was used to annotate the sequences of each genome.

### Analysis of *V. parahaemolyticus* genomic sequences

Based on the whole-genome single-nucleotide variations of the core genome extract using Snippy version 4.6.0 [22], the maximum likelihood method with the general time reversible model phylogenetic tree was constructed by FastTree version 2.1.10 [23] and finally visualized using the online tool iTOL [24]. A pan genome was calculated using Roary version 3.12.0 [25] with 80% minimum percentage identity for blastp. *Kaptive* databases for *V. parahaemolyticus* O- and K-antigen serotyping by running with *Kaptive* tool version 0.5.1 [26] were used to find ~52 kb region (between *dgkA* and *gtaB* genes) associated with the somatic synthesis locus (O-locus) and capsule synthesis locus (K-locus) for *V. parahaemolyticus* [1,27, 28]. Each strain’s O-locus and K-locus were extracted for comparing composition of genetic determinant region to reveal the relationship of different serotype strains by Easyfig version 2.2.2 [29]. To explore the possible sources of new serotype strains, blast [30] was used to search for genes of their special GI and O-locus. Furthermore, all *V. parahaemolyticus* genomic sequences available in GenBank until 5 June 2024 were downloaded, then selected with completeness >95% and contamination >5% by CheckM, as well as 333 * V*. *parahaemolyticus* isolated in this study to do cgMLST with the scheme from Pubmlst allele sequences by chewBBACA [31], then the phylogenetic tree was constructed.

## Results

### A novel pandemic serotype of *V. parahaemolyticus* (O10:K4) began to emerge in 2019 and quickly became the predominant strain in the Shanghai area

The O10:K4 serotype of *V. parahaemolyticus* was first detected in the Shanghai area in 2019 (it was isolated from a clinical sample collected on 6 June 2019 in Jinshan District, Shanghai). Subsequently, we aimed to trace whether the O10:K4 serotype was present in earlier samples to determine its earliest detection time; a retrospective study was conducted. It was found that among the 1,937 strains of *V. parahaemolyticu*s isolated from 2017 to 2018, the serological typing showed that the dominant serotype was O3:K6. The proportion of the O3:K6 serotype in 2017 and 2018 was 52.56% (513 strains) and 60.46% (581 strains), respectively, with no O10:K4 serotype identified (Table 2). By typing the *V. parahaemolyticus* strains isolated from 2019 to 2021, it revealed that the detection rate of the O10:K4 serotype in the 3-year period from 2019 to 2021 was 2.49% (22 strains), 17.78% (32 strains) and 69.58% (167 strains), respectively. By 2021, the O10:K4 serotype had surpassed the O3:K6 serotype to become the dominant serotype circulating in the Shanghai area (Table 2).

**Table 2.** Serotype and pandemic strains distribution of *V. parahaemolyticus* in Shanghai during 2017–2021

	2017		2018		2019		2020		2021	
Serotype	No. of isolation	No. of pandemic strains	No. of isolation	No. of pandemic strains	No. of isolation	No. of pandemic strains	No. of isolation	No. of pandemic strains	No. of isolation	No. of pandemic strains
**O10:K4**	**0 (0)**	**0 (0)**	**0 (0)**	**0 (0)**	22 (2.49)*	**22 (8.7)**	**32 (17.78)**	**32 (32.99)**	**167 (69.58)**	**167 (87.43)**
O3:K6	513 (52.56)	510 (90.27)	581 (60.46)	580 (86.7)	203 (52.45)	203 (80.24)	57 (31.67)	56 (57.73)	22 (9.17)	22 (11.52)
O4:KUT	194 (19.88)	0 (0)	103 (10.72)	0 (0)	44 (11.37)	0 (0)	25 (13.89)	0 (0)	14 (5.83)	0 (0)
O1:KUT	41 (4.2)	16 (2.83)	65 (6.76)	39 (5.83)	18 (4.65)	5 (1.98)	18 (10)	1 (1.03)	7 (2.92)	0 (0)
O4:K8	51 (5.23)	0 (0)	33 (3.43)	0 (0)	2 (0.52)	0 (0)	1 (0.56)	0 (0)	1 (0.42)	0 (0)
O3:KUT	19 (1.95)	6 (1.06)	10 (1.04)	6 (0.9)	29 (7.49)	6 (2.37)	8 (4.44)	3 (3.09)	3 (1.25)	1 (0.52)
O1:K56	8 (0.82)	0 (0)	16 (1.66)	0 (0)	3 (0.78)	0 (0)	5 (2.78)	0 (0)	5 (2.08)	0 (0)
O10:K60	24 (2.46)	24 (4.25)	10 (1.04)	10 (1.49)	2 (0.52)	2 (0.79)	0 (0)	0 (0)	0 (0)	0 (0)
O5:KUT	18 (1.84)	0 (0)	11 (1.14)	0 (0)	3 (0.78)	0 (0)	1 (0.56)	0 (0)	0 (0)	0 (0)
O4:K9	2 (0.2)	0 (0)	24 (2.5)	0 (0)	3 (0.78)	0 (0)	1 (0.56)	0 (0)	2 (0.83)	0 (0)
O10:KUT	19 (1.95)	0 (0)	6 (0.62)	2 (0.3)	1 (0.26)	0 (0)	4 (2.22)	1 (1.03)	1 (0.42)	1 (0.52)
O11:KUT	10 (1.02)	0 (0)	5 (0.52)	0 (0)	6 (1.55)	0 (0)	3 (1.67)	0 (0)	2 (0.83)	0 (0)
O1:K25	1 (0.1)	1 (0.18)	15 (1.56)	15 (2.24)	7 (1.81)	6 (2.37)	0 (0)	0 (0)	0 (0)	0 (0)
O8:KUT	6 (0.61)	0 (0)	14 (1.46)	0 (0)	2 (0.52)	0 (0)	0 (0)	0 (0)	0 (0)	0 (0)
O2:K3	10 (1.02)	0 (0)	7 (0.73)	0 (0)	1 (0.26)	0 (0)	2 (1.11)	0 (0)	1 (0.42)	0 (0)
O5:K64	3 (0.31)	3 (0.53)	10 (1.04)	9 (1.35)	0 (0)	0 (0)	3 (1.67)	3 (3.09)	0 (0)	0 (0)
O4:K4	7 (0.72)	0 (0)	4 (0.42)	0 (0)	1 (0.26)	0 (0)	1 (0.56)	0 (0)	1 (0.42)	0 (0)
O2:K28	2 (0.2)	0 (0)	4 (0.42)	0 (0)	2 (0.52)	0 (0)	4 (2.22)	0 (0)	1 (0.42)	0 (0)
O2:KUT	5 (0.51)	0 (0)	1 (0.1)	0 (0)	2 (0.52)	0 (0)	2 (1.11)	0 (0)	2 (0.83)	0 (0)
O1:K36	4 (0.41)	4 (0.71)	3 (0.31)	3 (0.45)	1 (0.26)	1 (0.4)	1 (0.56)	0 (0)	0 (0)	0 (0)
O5:K17	2 (0.2)	0 (0)	0 (0)	0 (0)	5 (1.29)	0 (0)	0 (0)	0 (0)	1 (0.42)	0 (0)
O4:K68	1 (0.1)	1 (0.18)	5 (0.52)	5 (0.75)	0 (0)	0 (0)	1 (0.56)	1 (1.03)	0 (0)	0 (0)
O3:K5	4 (0.41)	0 (0)	1 (0.1)	0 (0)	2 (0.52)	0 (0)	0 (0)	0 (0)	0 (0)	0 (0)
O4:K13	2 (0.2)	0 (0)	4 (0.42)	0 (0)	0 (0)	0 (0)	0 (0)	0 (0)	0 (0)	0 (0)
O3:K25	0 (0)	0 (0)	0 (0)	0 (0)	5 (1.29)	4 (1.58)	0 (0)	0 (0)	0 (0)	0 (0)
O1:K1	0 (0)	0 (0)	2 (0.21)	0 (0)	2 (0.52)	0 (0)	0 (0)	0 (0)	1 (0.42)	0 (0)
OUT:KUT	1 (0.1)	0 (0)	1 (0.1)	0 (0)	1 (0.26)	0 (0)	1 (0.56)	0 (0)	1 (0.42)	0 (0)
O1:K54	0 (0)	0 (0)	3 (0.31)	0 (0)	1 (0.26)	0 (0)	0 (0)	0 (0)	0 (0)	0 (0)
O2:K22	1 (0.1)	0 (0)	1 (0.1)	0 (0)	0 (0)	0 (0)	2 (1.11)	0 (0)	0 (0)	0 (0)
O3:K57	3 (0.31)	0 (0)	1 (0.1)	0 (0)	0 (0)	0 (0)	0 (0)	0 (0)	0 (0)	0 (0)
O4:K53	0 (0)	0 (0)	2 (0.21)	0 (0)	0 (0)	0 (0)	2 (1.11)	0 (0)	0 (0)	0 (0)
OUT:K8	4 (0.41)	0 (0)	0 (0)	0 (0)	0 (0)	0 (0)	0 (0)	0 (0)	0 (0)	0 (0)
O3:K44	0 (0)	0 (0)	0 (0)	0 (0)	3 (0.78)	3 (1.19)	0 (0)	0 (0)	0 (0)	0 (0)
O1:K20	0 (0)	0 (0)	0 (0)	0 (0)	1 (0.26)	0 (0)	1 (0.56)	0 (0)	1 (0.42)	0 (0)
O8:K21	0 (0)	0 (0)	1 (0.1)	0 (0)	1 (0.26)	0 (0)	0 (0)	0 (0)	1 (0.42)	0 (0)
O1:K38	2 (0.2)	0 (0)	1 (0.1)	0 (0)	0 (0)	0 (0)	0 (0)	0 (0)	0 (0)	0 (0)
O11:K20	1 (0.1)	0 (0)	0 (0)	0 (0)	0 (0)	0 (0)	2 (1.11)	0 (0)	0 (0)	0 (0)
O11:K5	0 (0)	0 (0)	3 (0.31)	0 (0)	0 (0)	0 (0)	0 (0)	0 (0)	0 (0)	0 (0)
Others†	18 (1.84)	0 (0)	14 (1.46)	0 (0)	14 (3.62)	1 (0.4)	3 (1.67)	0 (0)	6 (2.5)	0 (0)
Total	976 (100)	565 (100)	961 (100)	669 (100)	387 (100)	253 (100)	180 (100)	97 (100)	240 (100)	191 (100)

*In this study, 387 out of 884 *V*. *parahaemolyticus* strains were tested in 2019. This proportion was calculated based on the total number of 884 *V*. *parahaemolyticus* strains detected in 2019.

†Not pandemic strains include O4:K34, O6:K18, O8:K41, O3:K29, O3:K30, O4:K63, O11:K68, O3:K21, O3:K37, O3:K54, O4:K42, O5:K30, O3:K68, O11:K6, O3:K59, O3:K9, O1:K32, O1:K39, O1:K41, O1:K46, O1:K5, O1:K9, O10:K24, O10:K52, O11:K22, O11:K51, O3:K23, O3:K45, O3:K53, O3:K65, O3:K7, O3:K8, O4:K12, O4:K37, O4:K55, O5:K15, O5:K33, O6:K46, O6:KUT and O9:K46.

*V. parahaemolyticus* pandemic strains (*tdh+*, *toxRS-Mut+*) are highly pathogenic to humans. In the *V. parahaemolyticus* strains isolated in 2017–2019, the proportion of O3:K6 serotype in pandemic strains was 90.27% (510/565), 86.7% (580/669) and 80.24% (203/253), respectively. All the isolated *V. parahaemolyticus* O10:K4 serotype strains were identified as the pandemic strains. In the pandemic strains of *V. parahaemolyticus* detected in 2019, the serotype O3:K6 was the most prevalent, with 203 strains detected (accounting for 80.24%), followed by the serotype O10:K4, with 22 strains (accounting for 8.7%). In 2020, among the detected pandemic strains, O3:K6 remained the dominant serotype with 56 strains (accounting for 57.73%); the proportion of O10:K4 increased gradually with 32 strains (accounting for 32.99%) detected. In 2021, O10:K4 was detected in 167 strains (comprising 87.43%), having become the predominant serotype in the pandemic strains (Table 2).

### Co-existence of two distinct lineages (O10:K4_*α*_ and O10:K4_*β*_) in *V. parahaemolyticus* in Shanghai

In this study, genomic sequencing was conducted on 369 strains of *V. parahaemolyticus*, with 337 strains sourced from patients, 31 from food and 1 with an unclear source. A total of 221 pandemic strains and 148 non-pandemic strains were subjected to genomic sequencing. Among the pandemic strains, 220 strains were of the ST3 type, while 1 strain remained untyped. Non-pandemic strains were categorized into 49 different ST types, as well as some untyped (Table S1, available in the online Supplementary Material).

**Fig. 1.**
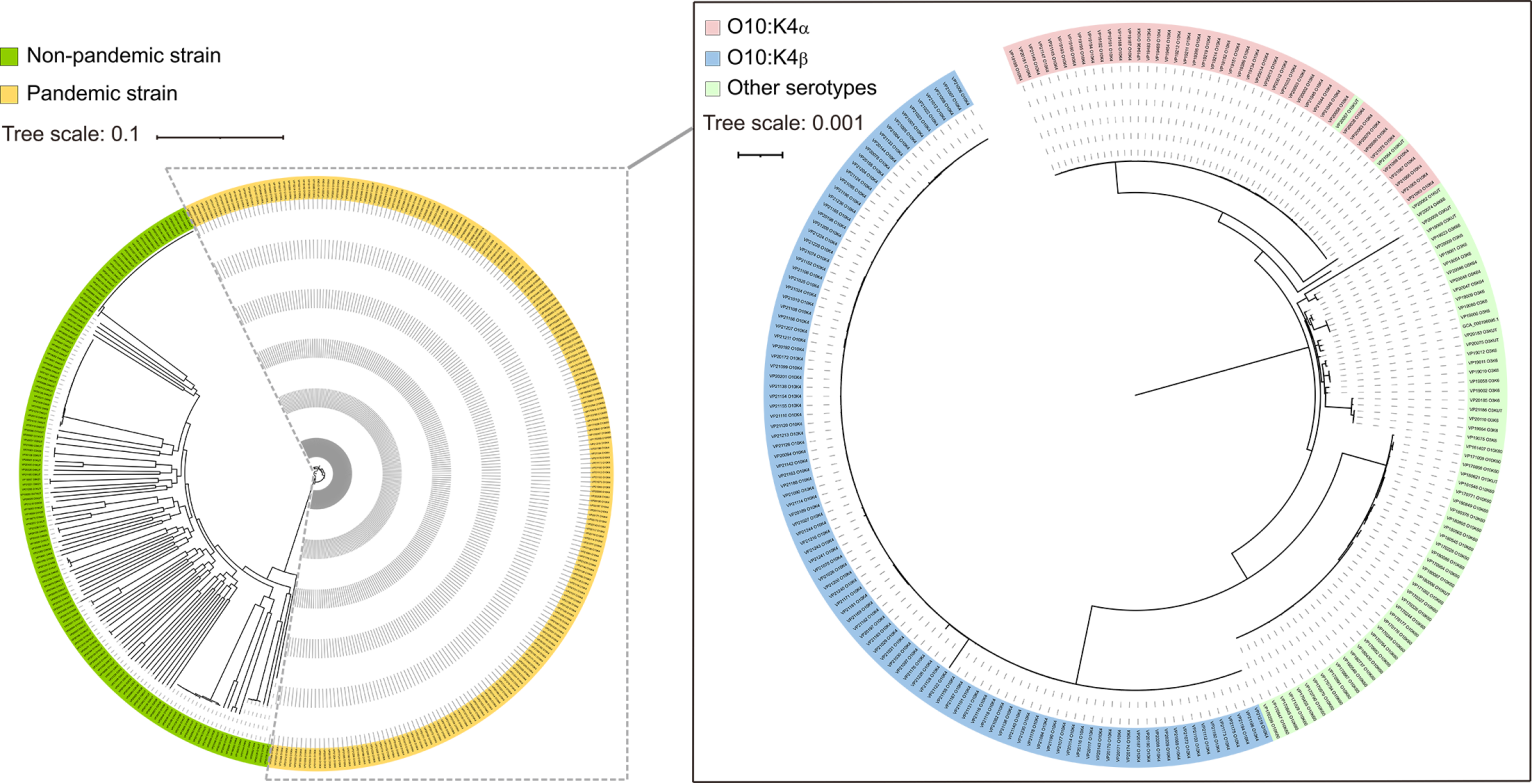
A maximum likelihood tree was constructed based on 369 genomes of *V. parahaemolyticus* strains. The phylogenetic tree was edited online using the website iTOL (https://itol.embl.de). The scale indicates the frequency of single-nucleotide variations.

**Fig. 2.**
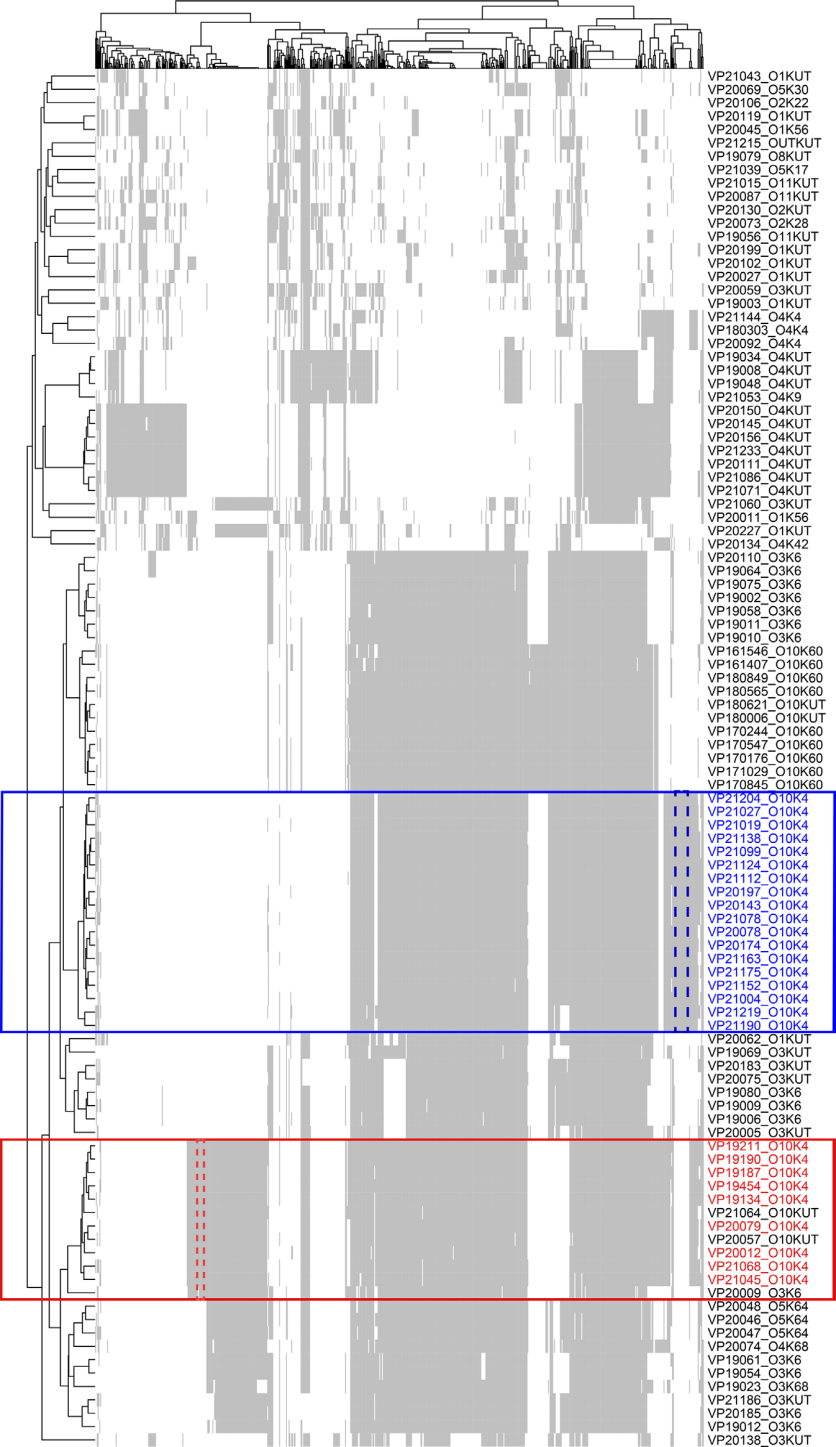
Pan-genome analysis of *V. parahaemolyticus*. Whole-genome sequencing data from 369 strains of *V. parahaemolyticus* were annotated using Prokka and subjected to pan-genome analysis with Roary to remove genes shared by >80% and genes present in <10%, resulting in the retention of 711 accessory genes for analysis. A total of 103 strains of *V. parahaemolyticus* with different serotypes were selected for visualization, with grey indicating the presence of the corresponding gene in the strain and white indicating the absence of the corresponding gene. Red text represents the O10:K4_*α*_ subtype strains, and the area enclosed by the red dashed line is the unique GI of the O10:K4_*α*_ subtype. Blue text represents the O10:K4_*β*_ subtype strains, and the area enclosed by the blue dashed line is the unique GI of the O10:K4_*β*_ subtype.

A total of 155 genomes of the O10:K4 serotype were obtained. Genomic annotation revealed that, with the exception of one strain that could not be typed by MLST, all remaining strains belonged to the ST3 type (Table S1). The phylogenetic tree constructed shows that 155 strains of O10:K4 serotype bacteria are distinctly divided into two genetic subtypes (O10:K4_*α*_ and O10:K4_*β*_) (Fig. 1). Among them, 46 strains belong to the O10:K4_*α*_ subtype, while 109 strains of *V. parahaemolyticus* belong to the O10:K4_*β*_ subtype. In addition, two pandemic strains of O10:K4_*α*_ subtype with non-typable K antigen O10:KUT (VP20057, VP21064). Pan-genome analysis further elucidates differences in the genomic composition between O10:K4_*α*_ and O10:K4_*β*_ (Fig. 2). The O10:K4_*α*_ subtype possesses a characteristic GI composed of eight genes, with a length of ~13 kb, which is also present in the three pandemic strains of O10:KUT (VP20057, VP21064) and O3:K6 (VP20009), but is not found in O10:K4_*β*_ and other strains of *V. parahaemolyticu*s. The sequences of this 13 kb long GI are completely identical in these 49 strains of *V. parahaemolyticus*. ATP-dependent RNA helicase RhlE gene was found in this GI, while the products encoded by the other genes are hypothetical proteins. blastn sequence alignment revealed a homologous sequence in *Vibrio vulnificus* (VV20-8B-2, Accession No. AP026552.1), with coverage of 100% and similarity of 97.11%, suggesting that this GI undergoes varying degrees of homologous recombination in the *Vibrio* genus (Table S2). The O10:K4_*β*_ subtype, on the other hand, possesses a GI composed of 17 genes, with a length of ~21 kb, all of which encode hypothetical proteins, and this GI is not found in other 260 *V*. *parahaemolyticus*. Interestingly, it was found that three genes from this GI were close to genes in a non-pandemic O2:KUT strain (VP21042), with similarity 86.30, 90.00, 85.60%, respectively (Table S3). The GI of the O10:K4_*β*_ subtype, as revealed by blastn sequence alignment, is identical to the previously reported O10:K4 serotype strains of *V. parahaemolyticus* (RMDVP1, Accession No. CP102434.1) isolated in Thailand [16]. blastn results also show a homologous sequence with 86% coverage and 95.16% similarity found in *Vibrio fluvialis* (2013V-1197, Accession No. CP051116.1) (Table S4).

**Fig. 3.**
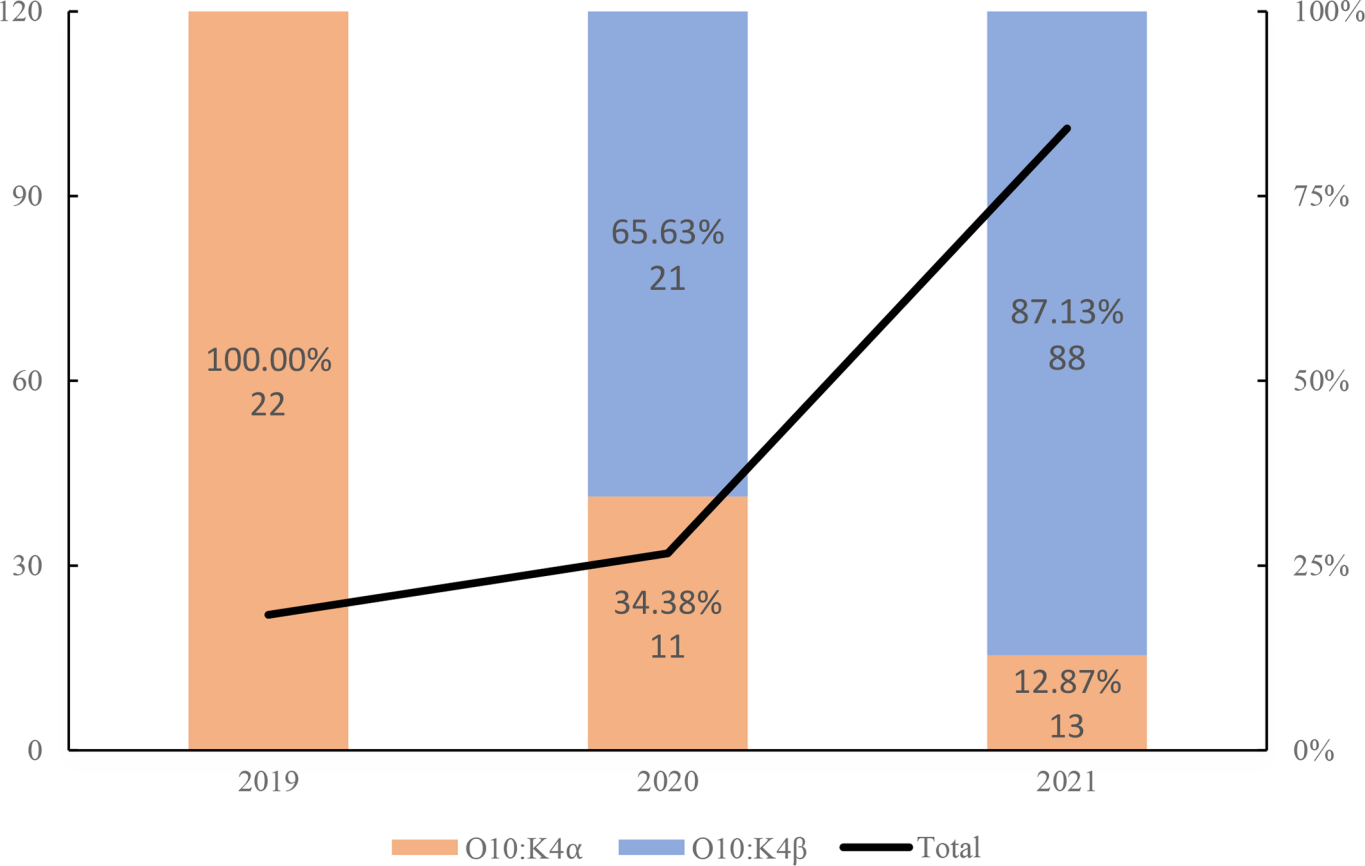
Distribution chart of the O10:K4_*α*_ and O10:K4_*β*_ subtype strains of *V. parahaemolyticus* isolated in Shanghai, China, from January 2019 to December 2021.

The detection rate of the O10:K4_*α*_ genetic subtype in O10:K4 strains in the Shanghai area decreased from 100% (22/22) in 2019 to 12.87% (13/101) in 2021, while the O10:K4_*β*_ subtype, which first appeared in 2020, increased from 65.62% (21/32) in its first detected year 2020 to 87.13% (88/101) in 2021 (Fig. 3). The carriage of the gene *orf8* in the strains of the two subtypes is also different. Among the 22 O10:K4_*α*_ strains isolated in 2019, 19 (86.36%) carried o*rf8* (Table 3). In 2020, all 21 strains of the O10:K4_*β*_ genetic subtype detected carried *orf8*, while among the 11 strains of the O10:K4_*α*_ genetic subtype detected, only 2 strains carried *orf8* (Table 3). By 2021, among the 101 O10:K4 serotype strains detected, there were 13 strains of the O10:K4_*α*_ genetic subtype, with only 1 strain (7.69%) carrying *orf8*, and among the 88 strains of the O10:K4_*β*_ genetic subtype, 78 strains (88.64%) carried *orf8*.

**Table 3.** Serotypes, virulence genes and ST of 155 O10:K4 *V. parahaemolyticus* strains

Serovar	Pandemic marker		Virulence gene		No. of isolate (%)			
	*orf8*	*toxRS-Mut*	*tdh*	*trh*	2019	2020	2021	2019–2021
O10:K4*_α_*	**+**	**+**	**+**	−	19 (86.36)	2 (18.18)	1 (7.69)	22 (47.83%)
	−	**+**	**+**	−	3 (13.64)	9 (81.82)	12 (92.31)	24 (52.17%)
O10:K4*_β_*	+	+	+	−	/	21 (100)	78 (88.64)	99 (90.83%)
	−	+	+	−	/	/	10 (11.36)	10 (9.17%)

### The O and K antigens of *V. parahaemolyticus* O10:K4 have different origins

**Fig. 4.**
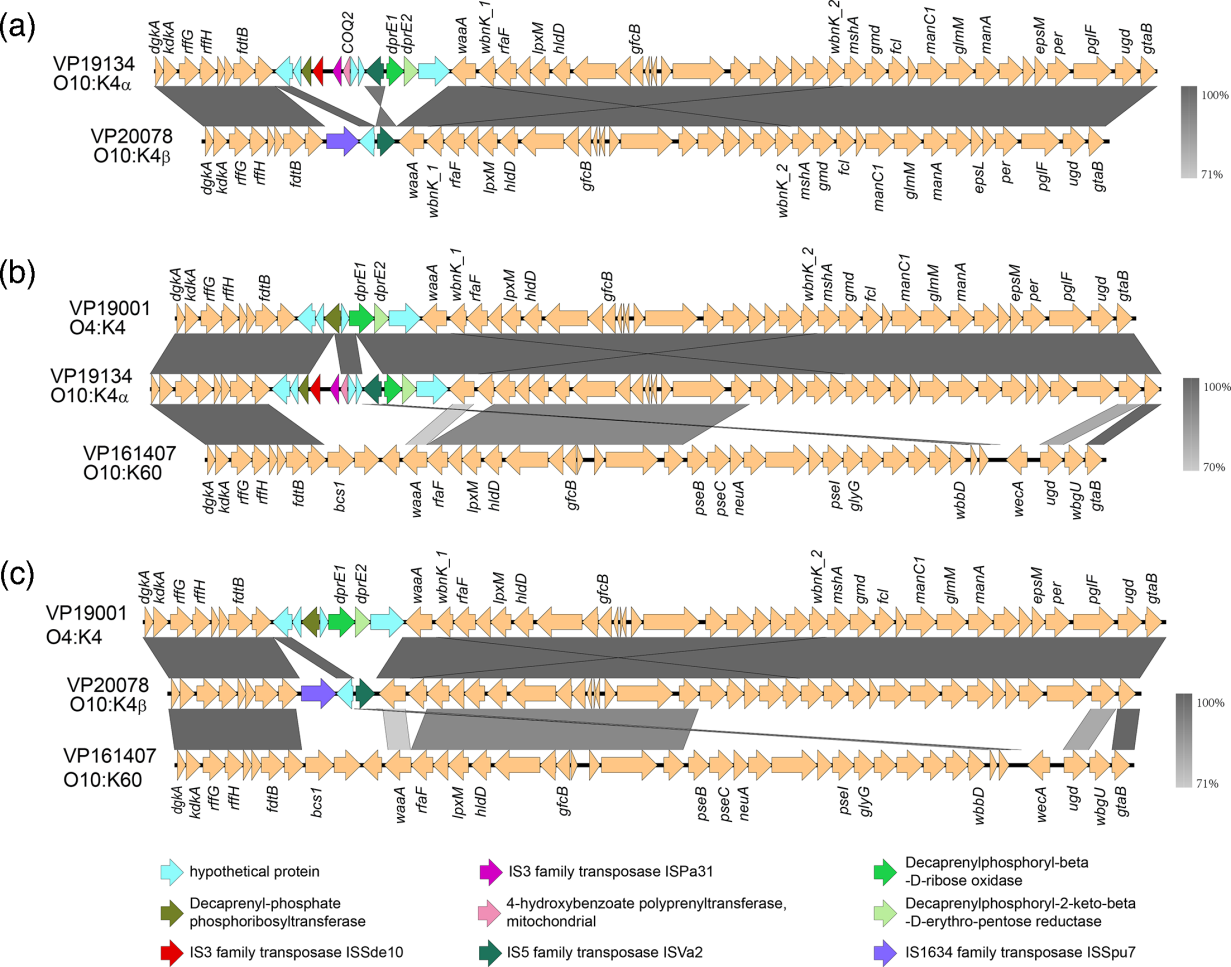
Structure and comparison diagram of the O and K antigen determination genes of O10:K4 serotype *V. parahaemolyticus*. (a) Comparison of O and K antigen determination genes between O10:K4_*α*_ (VP19134) and O10:K4_*β*_ (VP20078). (b) Comparison of the composition of O and K antigen determination regions of the O10:K4_*α*_ genetic subtype (VP19134) with the pandemic strain O10:K60 (VP161407) and the non-pandemic strain O4:K4 (VP19001). (c) Comparison of the composition of O and K antigen determination genes of the O10:K4_*β*_ genetic subtype (VP20078) with the pandemic strain O10:K60 (VP161407) and the non-pandemic strain O4:K4 (VP19001). The *Kaptive* tool was used to identify O and K antigen determination genes, and Easyfig was utilized to draw the antigen gene structure comparison chart. Arrows indicate the direction of the ORFs, different colours represent the corresponding protein products of the genes and the depth of the grey indicates the similarity of the nucleotide sequences. Between the genes *dgkA-hldD* is the O antigen determination region, and between the genes *hldD-gtaB* is the K antigen determination region.

The comparison of antigen gene structure showed that two subtypes of O10:K4 have the same source of K antigen but different sources of O antigen (Fig. 4a). The K antigen determinant region (*hldD-gtaB*) of O10:K4 strains has a consistent composition and high sequence similarity. The K antigen determinant region of O10:K4_*α*_ is consistent in gene composition with that of the K antigen of the O4:K4 strain (VP19001, a non-pandemic strain), with a total of 27 encoded genes, ~30 kb in length, suggesting that the K antigen of the new pandemic serotype variant comes from the non-pandemic strain of *V. parahaemolyticus* with the K4 antigen (Fig. 4b). The O10:K4_*β*_ genetic subtype is also consistent in gene composition with the K antigen of the O4:K4 strain (Fig. 4c).

The O antigen coding regions (*dgkA-hldD*) are different between O10:K4_*α*_ and O10:K4_*β*_, and both differ from known O antigen of O10:K60 strains, one of the frequently isolated pandemic variants. The O antigen coding regions of O10:K4_*α*_ and O10:K4_*β*_ have 11 totally different genes, which flank ISVa2 gene (IS5 family transposase). Gene insertions, deletions and inversions were observed in this region, suggesting that the O antigens of these two genetic subtypes have different origins. Besides the ISVa2 gene, IS3 family transposases ISSde10 and ISPa31 and other genes were also found in a 1.6 kb insertion segment in the O antigen determinant region of O10:K4_*α*_. The inserted fragment also contains the gene *COQ2* encoding 4-hydroxybenzoate polyprenyltransferase, mitochondrial, which is related to the oxidative metabolic chain, and two genes encoding hypothetical proteins may lead to changes in the O antigen structure. blastn search results show that these inserted genes exist in various *Vibrio* species other than *V. parahaemolyticus* (Table S5). Specifically, two of these inserted genes are not detected in *V. parahaemolyticus* but are found in the plasmid pVV20-8B-2 carried by *V. vulnificus* (VV20-8B-2, Accession No. AP026554.1), with 100% coverage and similarity over 96%.

Compared with the O4:K4 strain (VP19001), there is a 1 kb insertion sequence in O10:K4_*β*_ strains, encoding the IS5 family transposase ISVa2. Different from transposase ISVa2 in O10:K4_*α*_, blastn searching results show that this transposase has another origin. Homologous sequences of transposase ISVa2 in O10:K4_*β*_ were found in various *Vibrio* species, including *V. alginolyticus* (FDAARGOS_108, Accession No. CP014053.1) and in plasmids (pKp_SB612_1, Accession No. CP084831.1) carried by *Klebsiella pneumoniae* with 100% coverage and similarity >99% (Table S6). Another transposase (ISSpu7), belonging to the IS1634 family, was inserted into the O4 antigen determinant region, and it is also present in *V. alginolyticus* (2014V-1072, Accession No. CP046769.1), with 100% coverage and similarity. The O antigen coding sequencing of O10:K4_*β*_ is identical to that of *V. parahaemolyticus* strain RMDVP1 (Accession No. CP102434.1), a previously reported O10:K4 strain in Thailand, which hints that the variant from Thailand is the lineage of O10:K4_*β*_ [16].

### cgMLST analysis shows that O10:K4 is distributed in two clusters

The cgMLST phylogeny shows that O10:K4 serotype strains belong to pandemic strains and clearly divide into two clusters (cluster 1 and cluster 2) (Fig. S1, all strain information listed in Table S7). Cluster 1 contains 69 strains, and all strains are from China, including 46 O10:K4_*α*_ subtypes from this study and 2 pandemic strains of O10:KUT, as well as 18 O10:K4 strains detected in a food poisoning incident in Huzhou city, Zhejiang Province, China [32], and 3 O10:K4 strains detected in Jiaxing city, Zhejiang Province, China (Accession Nos. GCA_030028665.1, GCA_030028735.1, GCA_030028755.1). Cluster 2 includes 165 strains from 3 countries, and 109 are O10:K4_*β*_ subtype from this study, and 56 O10:K4 strains detected in Zhejiang Province [Jiaxing city (Accession Nos. GCA_030028705.1, GCA_030028745.1, GCA_030028765.1-GCA_030028915.1, GCA_030060495.1) and Huzhou city (Accession Nos. GCA_025792495.1, GCA_035063465.1, GCA_035063525.1)], Guangdong province (Accession Nos. GCA_024331565.1, GCA_024495085.1, GCA_024495165.1-GCA_024495385.1, GCA_024495585.1, GCA_024505245.1, GCA_024505265.1, GCA_024505365.1), Guangxi province (Accession Nos. GCA_019351375.1, GCA_023338245.1-GCA_023338635.1), Fengtai Region in Beijing (Accession Nos. GCA_034438875.1, GCA_034438935.1, GCA_034439095.1, GCA_034439135.1-GCA_034439255.1, GCA_034439315.1) China, also in Thailand (Accession No. GCA_028228685.1) and the USA (Accession Nos. GCA_023637745.1, GCA_024107635.1, GCA_028758665.1).

## Discussion

In the current study, an analysis of the serology and genomic characteristics of the newly emerged *V. parahaemolyticus* serovariant O10:K4 in Shanghai from 2019 to 2021 was conducted. In a comprehensive surveillance study conducted on the specimens submitted for analysis in the Shanghai region spanning the years 2017–2021, a significant diversity in serotypes was discerned. The serotype O3:K6 emerged as the most prevalent until the year 2020.

However, following the identification of the first O10:K4 strain in 2019, the detection rate of serotype O10:K4 has been increasing annually. Serotype O10:K4 has rapidly become the most prevalent in the Shanghai region. All detected O10:K4 serotype strains are pandemic clones with high pathogenicity (*tdh*+, *toxRS-Mut*+) and were isolated from clinical specimens (Table S1).

MLST analysis of 369 genomic sequences of the isolated *V. parahaemolyticus* strains showed that the ST3 type accounted for the highest proportion (221, 59.89%), all of which belong to the pandemic strain. Among the 155 O10:K4 serotype strains, 1 strain could not be typed due to the absence of a 12.5 kb gene fragment, including *pntA*, while the other 154 strains all belonged to the ST3 (Table S1).

Notably, pan-genome analysis revealed that each of these two genetic subtypes possesses characteristic but distinct GIs. The characteristic GI of the O10:K4_*β*_ subtype is present only in O10:K4_*β*_ strains. GI of the O10:K4_*α*_ subtype is also found in *V. parahaemolyticus* pandemic strains (serotypes O10:KUT and O3:K6) and *V. vulnificus*, which suggests that this particular GI may be horizontally transferred among *Vibrio* species.

In the comparative analysis of the antigenic determinant regions of O10:K4 strains, the K antigen determinant region sequences among all O10:K4 strains are highly similar or identical to those of serotype K4 strains, which hints at an exchange of K antigen among *V. parahaemolyticus*. However, the O antigen determinant regions of O10:K4 strains differ from those of the previous O10 group. For O10:K4_*α*_, it shows greater similarity to those of the O4 group strains with two insertions, but O10:K4_*β*_ has a totally new O antigen determinant sequence. This suggests two different origins of the O antigen of O10:K4_*α*_ and O10:K4_*β*_.

The presence of transposases facilitates the acquisition of exogenous gene fragments in *V. parahaemolyticus*. Comparative analysis of the O antigen determinant regions of the two genetic subtypes of O10:K4 revealed that both subtypes contain insertion sequences encoding the IS5 family transposase ISVa2; however, these two base sequences encoding these transposases derive differently, with significant differences in the sequence. Moreover, the O10:K4_*α*_ subtype encodes another transposase from the IS3 family, namely ISSde10 and ISPa31. The O10:K4_*β*_ subtype encodes ISSpu7 transposase of IS1634 family, which may account for the co-existence of these two genetic subtypes.

In addition, it was discovered that the pandemic strains O10:KUT (VP20057, VP21064) exhibit a high degree of sequence similarity with the O10:K4_*α*_ genetic subtype strains, which are located on the same branch of the evolutionary tree. The slide agglutination test can subtype both strains into the O10 serogroup, but the K antigen was untypeable due to an insertion in the K antigen determination sequence compared to the O10:K4_*α*_ strains. A 921 bp fragment inserted into the *pglF* gene (encoding UDP-*N*-acetyl-alpha-d-glucosamine C6 dehydratase), which encodes the IS5 family transposase ISVa2. Notably, the sequence of this fragment is identical to that of the inserted fragment in the O antigen region of serotype O10:K4_*α*_ with an opposite orientation but differs from that of O10:K4_*β*_, which may be caused by antigenic drift. This indicates that there is another serotype, such as O10:KUT, also genotypically part of the O10:K4_*α*_ genetic subtype.

Analysis of the isolation years for the two subtypes of O10:K4 strains reveals that the O10:K4_*α*_ genetic subtype is progressively decreasing, while the proportion of the O10:K4_*β*_ genetic subtype is consistently on the rise (Fig. 3). The cgMLST analysis results show that the O10:K4_*α*_ subtype is detected exclusively in China, whereas the O10:K4_*β*_ subtype has been identified in both the USA (Accession Nos. GCA_023637745.1, GCA_024107635.1, GCA_028758665.1) and Thailand (Accession No. GCA_028228685.1), demonstrating a more pronounced potential for regional dissemination. The exclusive detection of the O10:K4_*α*_ subtype in China does not indicate geographic restriction to China but rather reflects China’s strong detection capabilities. The high seafood consumption in Shanghai and surrounding regions, an important reservoir for *V. parahaemolyticus*, further boosts detection capacity. Although the isolation of *V. parahaemolyticus* is not technically demanding, Shanghai’s continuous and highly sensitive diarrhoeal disease surveillance system processes a large volume of samples annually. This high-throughput, sensitive system enabled the detection of the transient epidemic caused by the O10:K4_*α*_ subtype. Therefore, the identification of the O10:K4_*α*_ strain in this region may be attributed to these enhanced surveillance capabilities. In contrast, its potential presence in other areas might have gone undetected due to differences in surveillance intensity, sampling strategies or local seafood consumption patterns. Data from both the Shanghai region and the database underscore that, in comparison to the O10:K4_*α*_ subtype, the O10:K4_*β*_ subtype has a wider distribution, is becoming the predominant genetic subtype and continues to evolve.

This study suggests that the O10:K4_*β*_ subtype may pose a greater risk and provides a foundation for subsequent research on the evolution of pandemic strains of *V. parahaemolyticus*. It is very necessary to continue monitoring the prevalence of * V. parahaemolyticus*, including serotyping and genomic sequencing.

## Supplementary material

10.1099/mgen.0.001648 Uncited Supplementary Material 1.

10.1099/mgen.0.001648 Uncited Supplementary Material 2.

## Abbreviations

cgMLST: core genome multilocus sequence typing
GI: genomic island
LPS: lipopolysaccharide
