# Supplementary material for: Two different genotypes of Vibrio parahaemolyticus serotype O10:K4 have been co-circulating in Shanghai since 2020
Source: Microb Genom. 2026 Feb 16;12(2):001648. doi: 10.1099/mgen.0.001648 (PMC12921580; doi:10.1099/mgen.0.001648)
Supplement: Uncited Supplementary Material 1. [file mgen-12-01648-s001.pdf]

Supplementary Table 2. O10:K4$_{\alpha}$ VP19134 blastn results of the characteristic genomic island DNA sequences

Genes	Description	Scientific Name	Query Cover	Per. ident	Acc. Len	Accession	Matching Genes
VP19134_g 4219-4226	*Vibrio vulnificus* VV20-8B-2	Vibrio vulnificus	100%	97.11	3292876	AP026552.1	VV208B2_01140/01150/01160/01170/01180/01190/hsdR/01210
	Vibrio vulnificus strain 1908-10	Vibrio vulnificus	96%	97.31	3273700	CP118438.1	PVE41_10870/10875/10880/10885/10890/10895/10900/10905
	Vibrio parahaemolyticus strain HZ-52	Vibrio parahaemolyticus	89%	97.89	3471189	CP047985.1	GSS61_14435/14440/14445/14455/14460/14465
	Vibrio furnissii strain FDAARGOS_777	Vibrio furnissii	85%	97.45	3275692	CP040990.1	FIU11_02005/02010/02015/02020/02025/02030/02035
	Vibrio furnissii strain PartQ-Vfurnissii-RM8376	Vibrio furnissii	85%	97.55	3275680	CP064379.1	IUJ52_08940/08945/08950/08955/08960/08965/08970

Table note: The gene number annotated by Prokka. The matching genes refer to genes that correspond to the gene names in the "features" section of GenBank. /, the GenBank entry lacks the "features" section.

Supplementary Table 3. Three genes of O10:K4$_{\beta}$ GI compare with a non-pandemic strain VP21042

Gene of VP20078	Gene of VP21042	Similarity
VP20078_O10K4_04059	VP21042_O2KUT_04364	86.30%
VP20078_O10K4_04063	VP21042_O2KUT_04368	90.00%
VP20078_O10K4_04064	VP21042_O2KUT_04369	85.60%

Table note: The gene number annotated by Prokka, the similarity computing with MAGA.

Supplementary Table 4. O10:K4$_{\beta}$ VP20078 blastn results of the characteristic genomic island DNA sequences

Genes	Description	Scientific Name	Query Cover	Per. ident	Acc. Len	Accession	Matching Genes
VP20078_g4052-4068	*Vibrio parahaemolyticus* strain RMDVP1	*Vibrio parahaemolyticus*	100%	100	3300152	CP102434.1	RIMD4000701_20410/20420/20430/20440/20450/20460/20470/20480/20490/20500/20510/20520/20530/20540/20550/20560/20570
	Vibrio fluvialis strain 2013V-1197	*Vibrio fluvialis*	86%	95.19	3156804	CP051116.1	/
	Vibrio cholerae strain W10G	*Vibrio cholerae*	72%	94.06	3033188	CP053794.1	HPY12_12495/12500/12505/12510/12515/12520/12535/12540/12545/12550/12555/12560/12565
	Vibrio navarrensis strain 2014V-1106	*Vibrio navarrensis*	62%	96.09	3293562	CP051101.1	/

Table note: The gene number annotated by Prokka. The matching genes refer to genes that correspond to the gene names in the "features" section of

GenBank. /, the GenBank entry lacks the "features" section.

Supplementary Table 5. O10:K4$_{\alpha}$ VP19134 O antigenic determinant gene blastn results.

Genes	Product	Description	Scientific Name	Query Cover	Per. ident	Acc. Len	Accession	Matching Genes
VP19134_O10_g12	IS3 family transposase ISSde10	*Vibrio harveyi strain* fish8	*Vibrio harveyi*	100%	99.05	3597146	CP118594.1	/
		Vibrio fluvialis IDH05335	*Vibrio fluvialis*	100%	96.55	3364569	AP028128.1	KKIDH5335_07630
		Vibrio vulnificus VV20-8B-2 plasmid pVV20-8B-2	*Vibrio vulnificus*	100%	95.79	322993	AP026554.1	tnpA_9
		Vibrio sp. dhg plasmid unnamed1	Vibrio sp. dhg	100%	94.64	143489	CP028945.1	DBX26_24885
		Vibrio navarrensis strain 2540-90	*Vibrio navarrensis*	100%	94.44	3250151	CP061845.1	IF132_06320
		Vibrio natriegens strain PWH3a	*Vibrio natriegens*	100%	91.95	3332322	CP107282.1	OFO16_05640
		Vibrio anguillarum strain VIB12 plasmid p292	*Vibrio anguillarum*	100%	91.38	292095	CP023312.1	CMV05_22850
VP19134_O10_g13	IS3 family transposase ISPa31	*Vibrio harveyi* strain fish8	*Vibrio harveyi*	100%	99.54	3597146	CP118594.1	/
		Vibrio fluvialis IDH05335	*Vibrio fluvialis*	100%	99.09	3364569	AP028128.1	KKIDH5335_07590
		Vibrio vulnificus VV20-8B-2 plasmid pVV20-8B-2	*Vibrio vulnificus*	100%	98.17	322993	AP026554.1	VV208B2_46700
		Vibrio sp. dhg plasmid unnamed1	Vibrio sp. dhg	100%	95.66	143489	CP028945.1	DBX26_24885
		Vibrio navarrensis strain 08-2462	*Vibrio navarrensis*	100%	94.29	1278124	CP035681.1	/
		Vibrio tasmaniensis LMG 20012 plasmid p1 DNA	*Vibrio tasmaniensis*	100%	94.29	149865	AP025512.1	/
		Vibrio sp. J383 plasmid p.J383	Vibrio sp. J383	100%	94.29	201166	CP097295.1	M4S28_26210
		Vibrio sp. MAO6 plasmid pMAO6 DNA	Vibrio sp. MAO6	100%	91.55	299686	AP029601.1	MAO6_44130
VP19134_O10_g14	4-hydroxybenzoate	*Vibrio parahaemolyticus*	*Vibrio parahaemolyticus*	89%	100	100198	MK473648.1	/

	polyprenyltransferase, mitochondrial	strain G3556 clone K42_G3556						
		Vibrio parahaemolyticus strain G2879 clone K49_G2879	*Vibrio parahaemolyticus*	89%	100	137240	MK473641.1	/
VP19134_O10_g15	hypothetical protein	*Vibrio parahaemolyticus* strain G2879 clone K49_G2879	*Vibrio parahaemolyticus*	100%	99.75	137240	MK473641.1	/
		Vibrio parahaemolyticus strain 2012V-1116	*Vibrio parahaemolyticus*	100%	99.5	3383078	CP051113.1	/
		Vibrio parahaemolyticus strain G3556 clone K42_G3556	*Vibrio parahaemolyticus*	100%	99.5	100198	MK473648.1	/
VP19134_O10_g16	hypothetical protein	*Vibrio parahaemolyticus* strain VP16	*Vibrio parahaemolyticus*	93%	100	3288162	CP127846.1	QRT07_14180
		Vibrio parahaemolyticus strain 2012V-1116	*Vibrio parahaemolyticus*	93%	99.68	3383078	CP051113.1	/
VP19134_O10_g17	IS5 family transposase ISVa2	*Vibrio parahaemolyticus* strain Colony90	*Vibrio parahaemolyticus*	100%	97.83	1814246	CP078736.1	/
		Vibrio furnissii strain 2013V-1001	*Vibrio furnissii*	100%	98.05	3359696	CP046797.1	/
		Vibrio natriegens isolate	*Vibrio natriegens*	100%	97.94	3601552	OY781038.1	/
		Vibrio fluvialis strain ATCC 33809	*Vibrio fluvialis*	100%	97.72	1671895	CP014034.2	AL536_06165
		Vibrio parahaemolyticus strain Colony556	*Vibrio parahaemolyticus*	100%	97.5	1814246	CP078620.1	/
		Vibrio owensii strain SH14 plasmid pVHvo	*Vibrio owensii*	100%	97.39	69148	CP045861.1	APZ19_28510/28545
		Vibrio parahaemolyticus strain MVP1 plasmid pVa	*Vibrio parahaemolyticus*	100%	97.39	69047	CP043423.1	BSR23_026425/026450
		Vibrio campbellii strain LMB29 plasmid pVCON1	*Vibrio campbellii*	100%	97.39	70053	MH890610.1	/

Table note: The gene number annotated by Prokka. The matching genes refer to genes that correspond to the gene names in the "features" section of GenBank. /, the GenBank entry lacks the "features" section.

Supplementary Table 6. O10:K4$_{\beta}$ VP20078 O antigenic determinant gene blastn results

Genes	Product	Description	Scientific Name	Query Cover	Per. ident	Acc. Len	Accession	Matching Genes
VP20078_O10_g9	IS1634 family transposase ISSpu7	*Vibrio parahaemolyticus* strain RMDVP1	*Vibrio parahaemolyticus*	100%	100	3300152	CP102434.1	NR793_22675
		Vibrio alginolyticus strain FDAARGOS_108	*Vibrio alginolyticus*	100%	99.88	3418310	CP014053.1	AL539_21630
		Vibrio rumoiensis FERM P-14531	*Vibrio rumoiensis*	100%	99.7	1169909	AP018686.1	/
		Aeromonas caviae GSH8M-1 plasmid pGSH8M-1-1	*Aeromonas caviae*	100%	99.58	153814	AP019196.1	ACGSH8M1_p10900
		Providencia heimbachae strain NCTC12003	*Providencia heimbachae*	100%	99.58	4286000	LS483422.1	NCTC12003_00707
		Klebsiella pneumoniae strain SB612 plasmid pKp_SB612_2	*Klebsiella pneumoniae*	100%	99.58	131535	CP084832.1	LGM16_26920
		Klebsiella pneumoniae strain SB612 plasmid pKp_SB612_1	*Klebsiella pneumoniae*	100%	99.58	172669	CP084831.1	LGM16_26390
		Klebsiella pneumoniae strain SB612 chromosome	*Klebsiella pneumoniae*	100%	99.58	5324939	CP084830.1	LGM16_00765
		Aeromonas veronii plasmid pWP8-W19-CRE-03_1	*Aeromonas veronii*	100%	99.58	166424	AP022282.1	WP8W19C03_P10740
		Aeromonas veronii DNA, strain: WP8-W19-CRE-03	*Aeromonas veronii*	100%	99.58	4555339	AP022281.1	WP8W19C03_00560/17380
		Aeromonas caviae plasmid pWP8-S18-CRE-01_1	*Aeromonas caviae*	100%	99.58	153814	AP022242.1	WP8S18C01_P10690
		Vibrio zhugei strain HBUAS61001	*Vibrio zhugei*	100%	98.86	2901449	CP033078.1	/
		Vibrio cidicii strain 2756-81	*Vibrio cidicii*	100%	94.12	3431466	CP046804.1	/

		Vibrio parahaemolyticus strain RMDVP1	*Vibrio parahaemolyticus*	100%	100	3300152	CP102434.1	NR793_22670
		Vibrio parahaemolyticus strain G3556 clone K42_G3556	*Vibrio parahaemolyticus*	97%	99.87	100198	MK473648.1	/
VP20078_O10_g10	hypothetical protein	*Vibrio parahaemolyticus* strain BM26A	*Vibrio parahaemolyticus*	97%	99.49	3378882	CP141290.1	/
		Vibrio parahaemolyticus strain G2879 clone K49_G2879	*Vibrio parahaemolyticus*	97%	99.23	137240	MK473641.1	/
VP20078_O10_g11	IS5 family transposase ISVa2	*Vibrio alginolyticus* strain 2014V-1072	*Vibrio alginolyticus*	100%	100	3456677	CP046769.1	/
		Vibrio parahaemolyticus strain Vb2289 plasmid pVb2289-tet	*Vibrio parahaemolyticus*	100%	100	280017	OQ622012.1	WKV20138.1
		Vibrio fluvialis IDH05335 plasmid pIDH5335-1	*Vibrio fluvialis*	100%	100	370014	AP028130.1	KKIDH5335_46370/46890/47420/47010/47160
		Vibrio fluvialis IDH05335	*Vibrio fluvialis*	100%	100	3364569	AP028128.1	KKIDH5335_03850
		Vibrio parahaemolyticus strain RMDVP1	*Vibrio parahaemolyticus*	100%	100	3300152	CP102434.1	NR793_22665
		Vibrio parahaemolyticus strain 20151116002-3 plasmid pVPGX2015-2	*Vibrio parahaemolyticus*	100%	99.67	69128	CP034308.1	EHC68_26915/26945
		Vibrio parahaemolyticus strain 20151116002-3	*Vibrio parahaemolyticus*	100%	99.67	1886547	CP034306.1	EHC68_18600/19740/26290/26395/26640/17720
		Vibrio parahaemolyticus strain 20160303005-1 plasmid pVPSD2016-5	*Vibrio parahaemolyticus*	100%	99.67	74488	CP034304.1	EHC69_29565/29595

	Vibrio owensii strain SH14 plasmid pVHvo	*Vibrio owensii*	100%	99.67	69148	CP045861.1	APZ19_28510/28545
	Vibrio owensii strain SH14	*Vibrio owensii*	100%	99.67	3689702	CP045859.1	APZ19_06855
	Vibrio parahaemolyticus strain MVP1 plasmid pVa	*Vibrio parahaemolyticus*	100%	99.67	69047	CP043423.1	BSR23_026425/026450
	Vibrio parahaemolyticus strain MVP1	*Vibrio parahaemolyticus*	100%	99.67	3446010	CP043421.1	BSR23_004335/008480/010465
	Vibrio campbellii strain LMB29 plasmid pVCON1	*Vibrio campbellii*	100%	99.67	70053	MH890610.1	/
	Vibrio owensii strain 1700302 plasmid pVOWZ2	*Vibrio owensii*	100%	99.67	68834	CP033140.1	D0812_29935/29965

Table note: The gene number annotated by Prokka. The matching genes refer to genes that correspond to the gene names in the "features" section of GenBank. /, the GenBank entry lacks the "features" section.

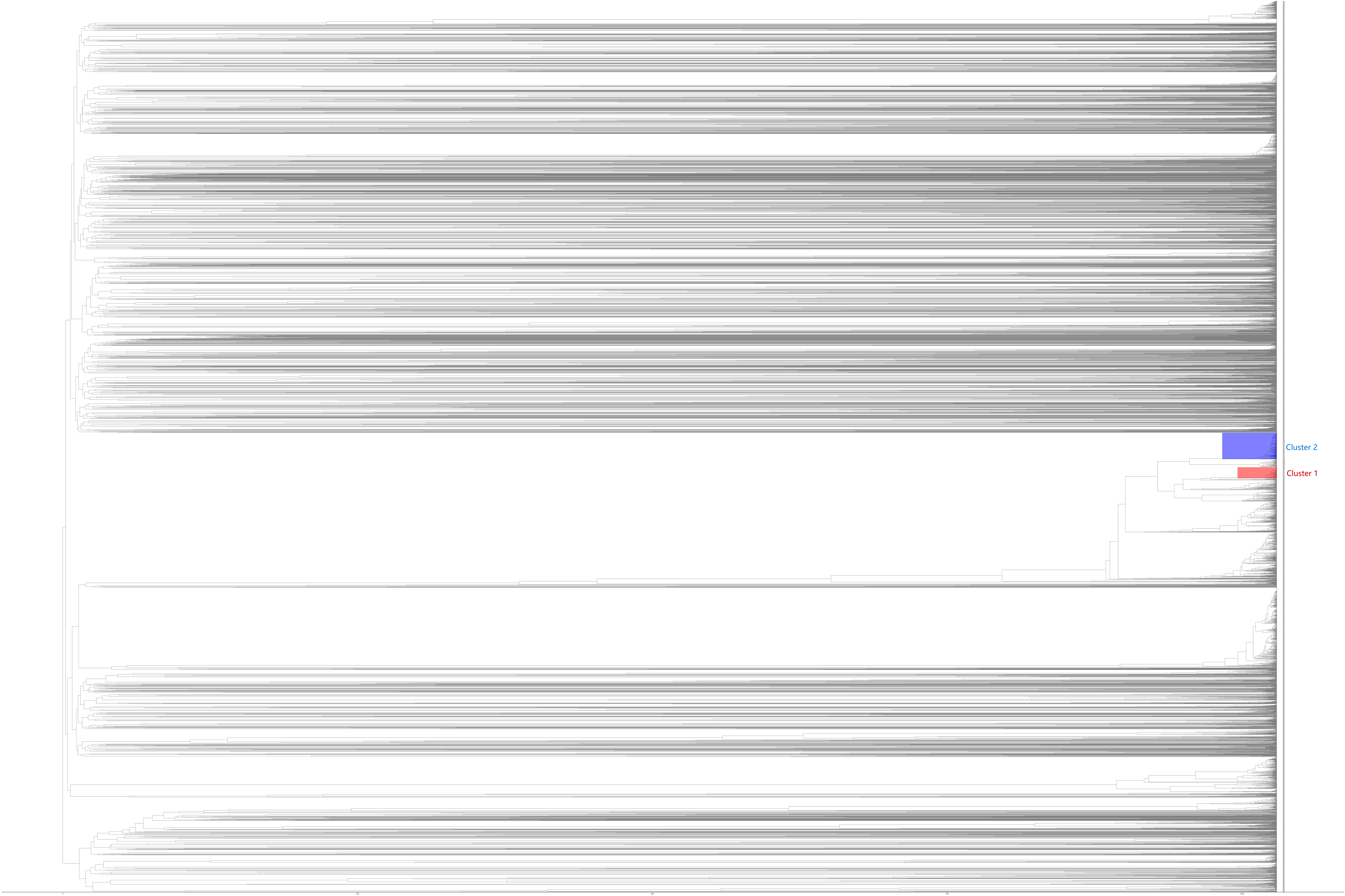

Cluster 2
Cluster 1
0
250
500
750
1000
